# Breast cancer dataset with biomarker Biglycan

**DOI:** 10.1016/j.dib.2023.108978

**Published:** 2023-02-14

**Authors:** Pedro Clarindo da Silva Neto, Rafael Kunst, Jorge Luis Victoria Barbosa, Ana Paula Thiesen Leindecker, Ricardo F. Savaris

**Affiliations:** a Universidade do Vale do Rio dos Sinos (UNISINOS), Av. Unisinos, 950 - Cristo Rei, São Leopoldo, 93022-750, RS, Brazil; b Instituto Federal de Educação, Ciência e Tecnologia de Mato Grosso (IFMT), Rua, Zulmira Canavarros, 95 - Centro Cuiabá, 78605-000, MT, Brazil; c Universidade Federal do Rio Grande do Sul / Porgrama de Pós-graduação em Medicina: Ciências Cirúrgicas, Rua Ramiro Barcelos 2300 - Santa Cecilia Porto Alegre, 90035-007, RS, Brazil

**Keywords:** Breast cancer, Biomarker, Biglycan, Images dataset

## Abstract

This dataset is composed of photomicrographs of the immunohistochemical expression of Biglycan (BGN) in breast tissue, with and without cancer, using only the staining of 3-3′ diaminobenzidine (DAB), after processing images with color deconvolution plugin, from Image J. The immunohistochemical DAB expression of BGN was obtained using the monoclonal antibody (M01) (clone 4E1-1G7 - Abnova Corporation, mouse anti-human). Photomicrographs were obtained, under standard conditions, using an optical microscope, with UPlanFI 100x objective (resolution: 2.75 mm), yielding an image size of 4800 × 3600 pixels. After color deconvolution, the dataset with 336 images was divided into 2 two categories: (I) with cancer and (II) without cancer. This dataset allows the training and validation of machine learning models to diagnose, recognize and classify the presence of breast cancer, using the intensity of the colors of the BGN.

**Specifications Table**

**Table:** 

Subject	Computer Science, Health and medical sciences, Biological sciences
Specific subject area	Image processing
Type of data	Raw
How the data were acquired	The dataset consists of images from histological sections with and without breast cancer tumors. The expression of the BGN protein was obtained by an immunohistochemical technique using the monoclonal BGN antibody (M01) (clone 4E1-1G7 - Abnova Corporation, mouse anti-human). next, images were processed using color deconvolution, a plugin from ImageJ and only DAB images were selected.
Data format	The images are in PNG format with a standard size of 128 × 128 pixels.
Description of data collection	The images were obtained with an optical microscope (Olympus BX51 microscope; Olympus Optical Co., Tokyo, Japan) connected to a digital color camera (Olympus DP73; OM Digital Solutions Co., Tokyo, Japan). Images were obtained using an UPlanFI 100x; oil immersion objective, with a resolution: 2.75 mm (Olympus), with an original size of 4800 × 3600 pixels, under standard conditions.
Data source location	All photos derived from 3 µm sections from Formalin-Fixed Paraffin-Embedded (FFPE) tissue blocks, were obtained from the pathological archive of Hospital de Clínicas in Porto Alegre, Brazil.
Data accessibility	The images are available online on the Mendeley Data website. Repository name: Biglycan breast cancer dataset. Direct link to the dataset: https://data.mendeley.com/datasets/mprsccwxb7/3 Data identification number (permanent identifier, i.e. DOI number): http://dx.doi.org/10.17632/mprsccwxb7.3

## Value of the Data

• The data provide of histological images of breast tissue with and without cancer with the expression of the Biglycan biomarker, through the intensity of the staining of 3-3′ diaminobenzidine (DAB), using the process of color deconvolution.
• Researchers belonging to different areas can benefit from this dataset. Computer scientists and data scientists can benefit from the data provided to train and evaluate machine learning and deep learning models for various purposes such as recognition of breast cancer tissue based on BGN-DAB expression, a possible biomarker for this condition. Pathologists and other healthcare professionals can use the dataset and models generated from the dataset to deal with potential diseases.
• This dataset can potentially impact society, as it allows the concept of differentiation, classification and prediction models based on the expression of a possible biomarker for cancer, as Biglycan [1]. Image dataset and artificial intelligence algorithms optimize the analysis process, positively impacting disease prediction. Automated diagnosis is essential to prevent and control diseases [2].

## 1 Objective

The purpose of capturing the images and performing the color deconvolution process is to have a dataset formed from these histological images with the biomarker Biglycan, to provide a study of the difference between the expression of the biomarker between tissues with and without breast cancer.

## 2 Data Description

The dataset consists of two folders with a total of 336 images. The first and second folder contains images of breast tissue with and without cancer, respectively. Immunohistochemical expression analysis was performed independently using ImageJ and manual identification of regions of interest. All histological results were confirmed by a board certified pathologist to confirm the diagnosis.

## 3 Experimental Design, Materials and Methods

The dataset was constructed from pathology slides derived by the pathology service of the Hospital de Clínicas de Porto Alegre (HCPA); composed images were from histological sections stained with Liquid DAB+Substrate Chromogen System (K3468, Dako, DK-2600 Glostrup, Denmark) according to the manufacturer's instructions using 3,3′-diaminobenzidine (DAB) as chromogen. Slides, with and without breast cancer tumor, were photographed using an optical microscope (Olympus BX51 microscope; Olympus Optical Co., Tokyo, Japan) connected to a digital color camera (Olympus DP73; OM Digital Solutions Co., Tokyo, Japan). Images were obtained using a 100x objective (UPLFL 100X; Oil Immersion, Olympus).

This study was initially approved by the Research Ethics Committee of Hospital de Clínicas de Porto Alegre (2019/0337) CAAE 15329119.9.0000.5327 with the objective to verify the expression of BGN in breast tissues with and without cancer, using the D-HSCORE method [3]. The Research Ethics Committee waived the Informed Consent Form because the breast samples were older than 5 years.

**Fig. 1:**
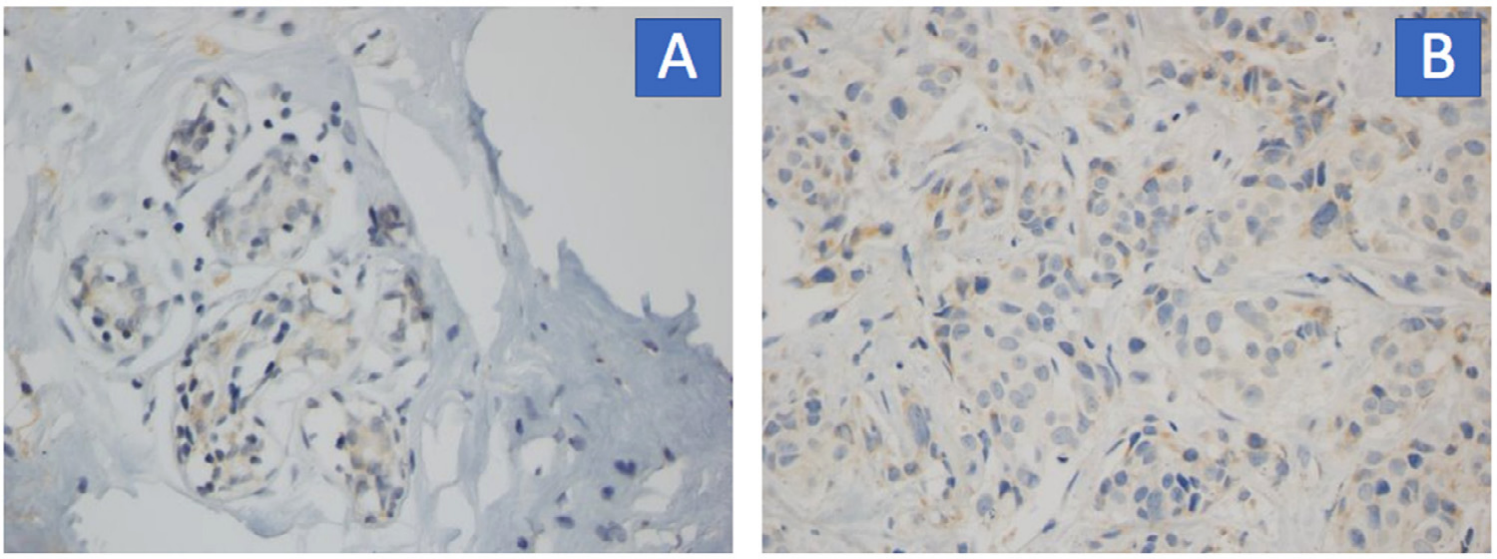
Example of images labeled by pathologists: **A** - normal breast tissue and **B** - breast cancer.

Images were labeled as cancer and healthy (cancer-free) by health researchers. Fig. 1 depicts representative images of the dataset.

Initially, researchers of the project performed the color deconvolution process manually for each image. The original photomicrograph was split into 3 different images: background, DAB and hematoxylin. In this case, immunoexpression of BGN, using 3-3′ diaminobenzidine (DAB) as a chromogen, was used. DAB acts as a chromogen, which, in the presence of the peroxidase enzyme, produces a brown precipitate, revealing the immunostained tissues. DAB immunostaining had different intensities, reflecting the expression of the BGN protein [4].

**Fig. 2:**
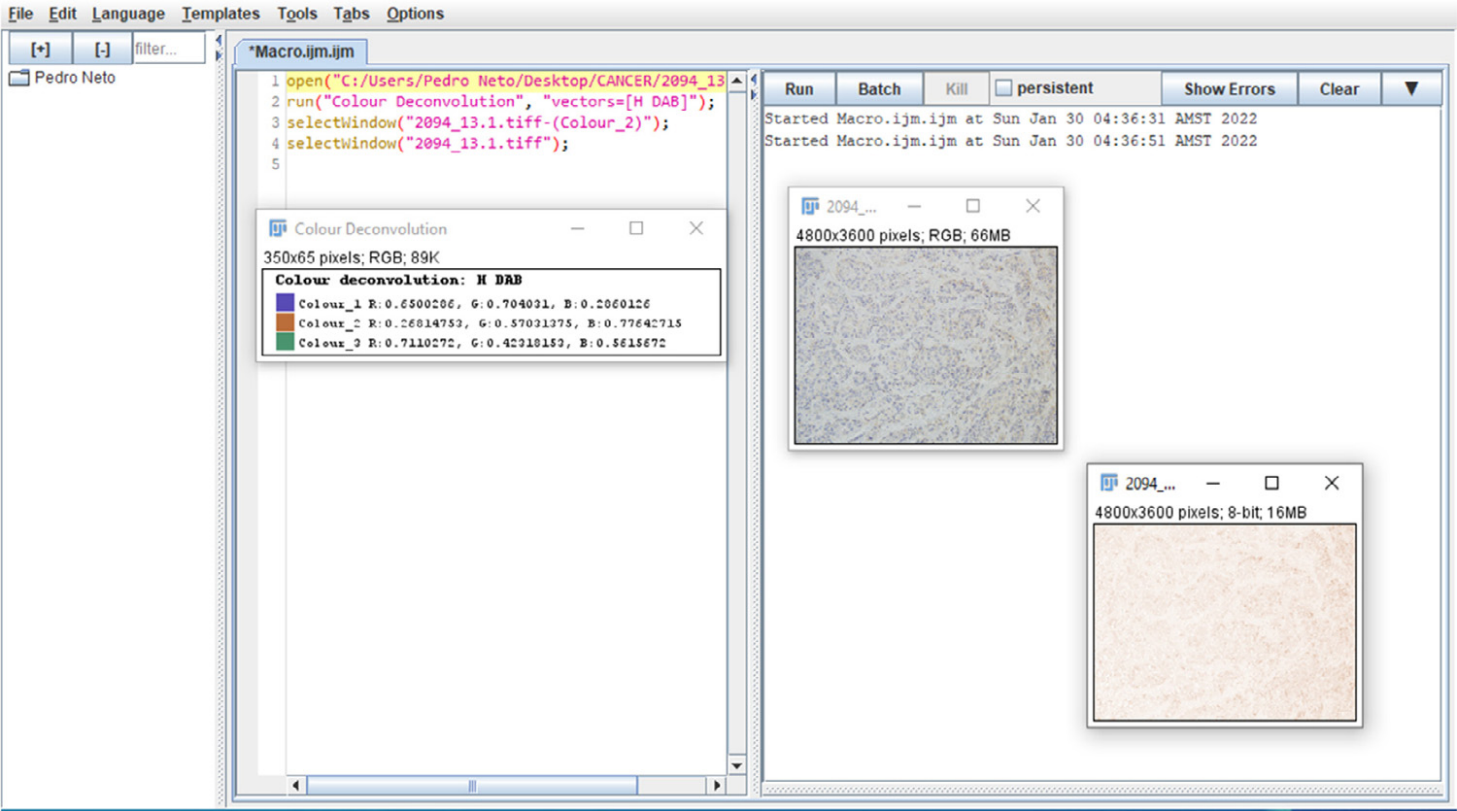
Execution process of the color deconvolution script in the ImageJ software.

This manual step proved to be time consuming. To increase the performance of this step, a script was created, through macro capture, so that the Fiji software performed, in an automated way: the staining using hematoxylin and DAB (HDAB), a resource available by the software in the color deconvolution option, as shown in Fig. 2. Herein, the whole process was carried out just by indicating the directory of the original images and the directory where the images should be stored after the color deconvolution process (Table 1).

**Table 1:** describes the type of breast tissue and the number of images per folder.

Folder	Number of images
Cancer	203
Healthy	133
**Total number of images**	336

## Ethics Statements

We the authors consciously assure that for the article “Breast cancer dataset with biomarker Biglycan” the following is fulfilled:

1. This article is the authors’ own original work, which has not been previously published elsewhere;
2. The dataset article is not currently being considered for publication elsewhere;
3. The article reflects the authors’ own research and analysis in a truthful and complete manner;
4. The article properly credits the meaningful contributions of co-authors;
5. All authors have been personally and actively involved in substantial work leading to the article and will take public responsibility for its content.

We agree with the above statements and declare that this submission follows the policies of Data in Brief as outlined in the Guide for Authors and in the Ethical Statement.

## CRediT Author Statement

**Pedro Clarindo da Silva Neto:** Conceptualization, Writing – original draft, Software; **Rafael Kunst:** Writing – review & editing, Supervision; **Jorge Luis Victoria Barbosa** and **Ricardo F. Savaris:** Writing – review & editing; **Ana Paula Thiesen Leindecker:** Validation, Data curation.

## Declaration of Competing Interest

The authors declare that they have no known competing financial interests or personal relationships that could have appeared to influence the work reported in this paper.

## Data Availability

Biglycan breast cancer dataset (Original data) (Mendeley Data).
